# Non-Descriptive

**Published:** 1909-10

**Authors:** 


NON-DESCRIPTIVE.—The general practitioner is frequently called upon to prescribe for women whose ailment is non-descriptive; they are depressed, have forebodings, and feel that their entire system is out of order, nerves completely unstrung, pains, aches and a general feeling of lassitude and debility. The physician is unable to perceive any positive symptoms indicating other than a normal condition, although he realizes something must be done to satisfy his patient until he is able to locate the cause. In such non-descriptive cases, Dioviburnia combined with Neurosine (equal parts) will usually after a week or ten days give entire relief. Dose, tablespoonful in wine glass of water three times a day.

No detrimental after effects need be feared in administering Dioviburnia and Neurosine, as neither contain opium, morphine, chloral or other deleterious drugs. (Any physician can obtain formula by writing the manufacturers.)

